# A Rare Case of Endobronchial Metastasis of Uterine Leiomyosarcoma Presenting as Bilateral Spontaneous Pneumothorax

**DOI:** 10.7759/cureus.95837

**Published:** 2025-10-31

**Authors:** Jaytin Gupta, Akanksha Girish, Ihab Masri, Jessica S Wang-Memoli, Ahmad Obeidat

**Affiliations:** 1 Department of Internal Medicine, NewYork-Presbyterian, Weill Cornell Medical Center, New York City, USA; 2 Department of Surgery, Stamford Hospital, Stamford, USA; 3 Department of Internal Medicine, MedStar Washington Hospital Center, Washington, USA; 4 Department of Pulmonary and Critical Care Medicine, MedStar Washington Hospital Center, Washington, USA; 5 Department of Internal Medicine, Washington University School of Medicine, St. Louis, USA

**Keywords:** endobronchial metastasis, lung metastasis, pneumothorax, thoracic oncology, uterine leimyosarcoma

## Abstract

This case report describes a rare presentation of uterine leiomyosarcoma (LMS) with endobronchial metastasis (EBM) manifesting as bilateral pneumothorax. A 48-year-old woman with a history of uterine LMS and lung metastasis presented with dyspnea and was diagnosed with EBM obstructing the left upper lobe. Despite treatment, the patient experienced progressive respiratory failure and succumbed to the disease. This case highlights the aggressive nature of EBM in LMS and emphasizes the importance of considering this rare complication in patients with LMS and respiratory symptoms such as dyspnea and cough without hemoptysis.

## Introduction

Uterine leiomyosarcoma (LMS), an aggressive cancer with high recurrence rates, typically spreads hematogenously and rarely via lymphatics. Uterine LMS represents 25-36% of uterine sarcomas and 1% of all uterine malignancies. The annual incidence rate is less than two per 100,000 women, with a median age of diagnosis of 56 years [1]. Even early-stage uterine LMS is characterized by high rates of recurrence and metastasis, ranging from 53% to 71% [1]. Despite the rarity of uterine sarcomas, constituting just 3.2% of all invasive uterine cancers, LMS has a predilection for distant metastasis to the lungs [2]. Endobronchial metastasis (EBM) from uterine LMS is exceptionally rare, with only a few isolated case reports in the literature. EBM most commonly arises from breast, colorectal, and renal carcinomas but is not typical of uterine LMS. Our case underscores the exceptional nature of EBM in LMS, presenting as spontaneous pneumothorax (SPTX), a clinical scenario seldom documented in medical literature. Through this report, we aim to shed light on diagnostic challenges and clinical implications of such an unusual metastatic pattern.

This article was presented at the 2023 CHEST Annual Meetings at the Airway Tumors Session on October 9, 2023. 

## Case presentation

**Figure 1:**
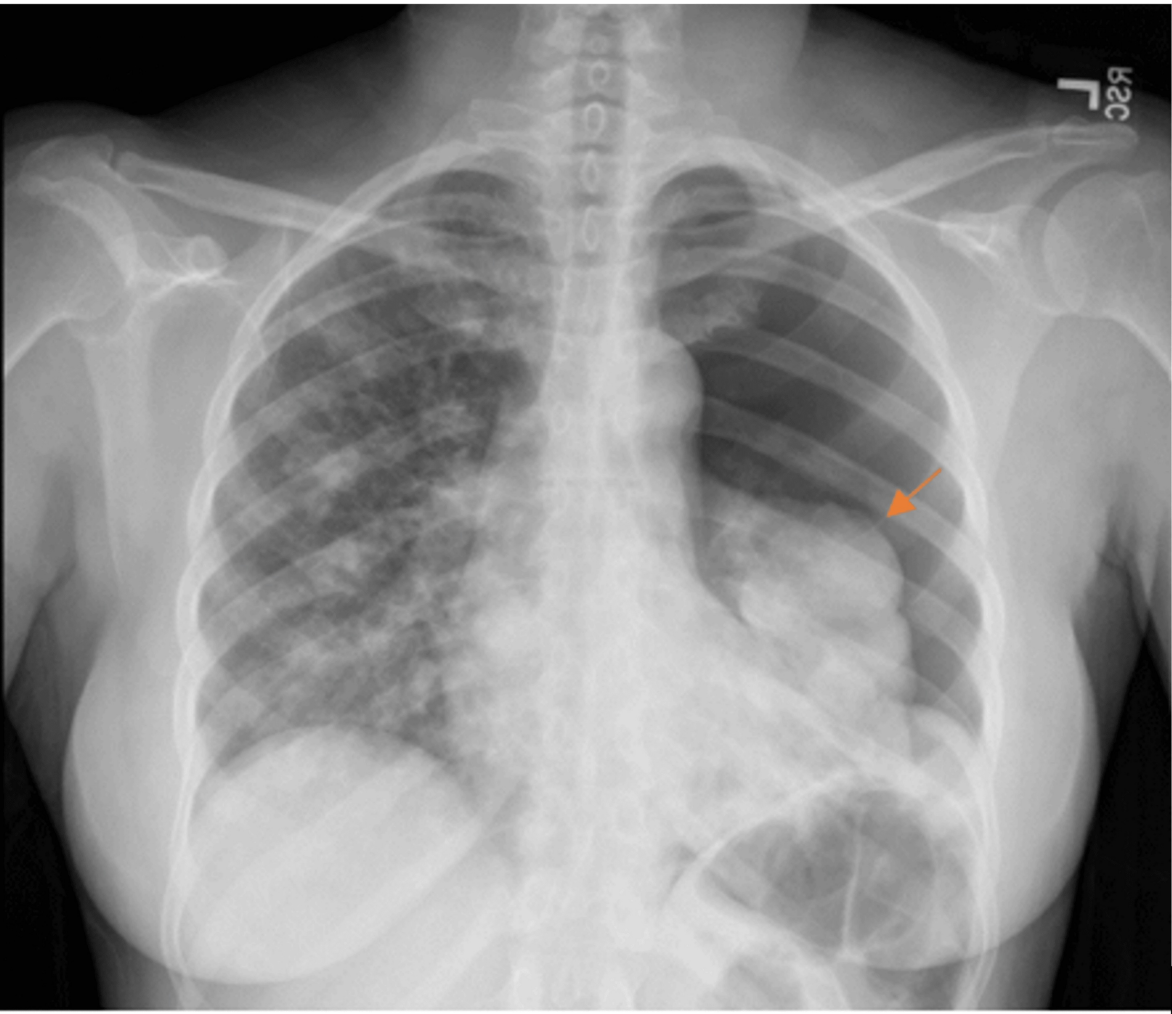
Anteroposterior (AP) chest radiograph demonstrating left PTX and mild right PTX. The orange arrow indicates the site of pulmonary metastasis with overlying PTX in the left lung. PTX: Pneumothorax

A 48-year-old woman presented with shortness of breath for four days. X-ray on presentation demonstrated left PTX (Figure 1), following which interventional pulmonology was engaged for placement of a 14 French left chest tube thoracostomy. The patient also developed a small right PTX seen on chest X-ray (CXR) for which a subsequent 12 French right chest tube thoracostomy was planned (Figure 1).

**Figure 2:**
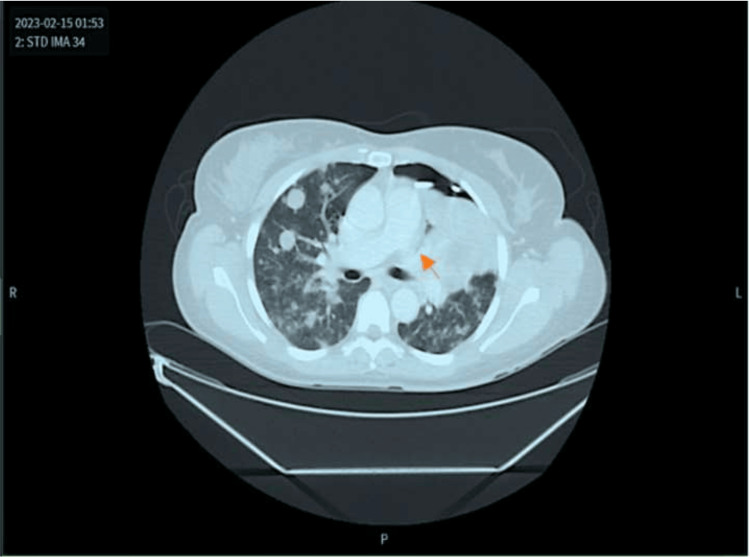
Axial-plane CT with coronal reconstruction (orange arrow) revealed complete left upper lobe collapse.

The orange arrow in the chest computed tomography (CT) scan demonstrates complete left upper lobe (LUL) collapse with persistent PTX and multiple bilateral metastatic nodules progressing from recent prior imaging studies (Figure 2). 

**Figure 3:**
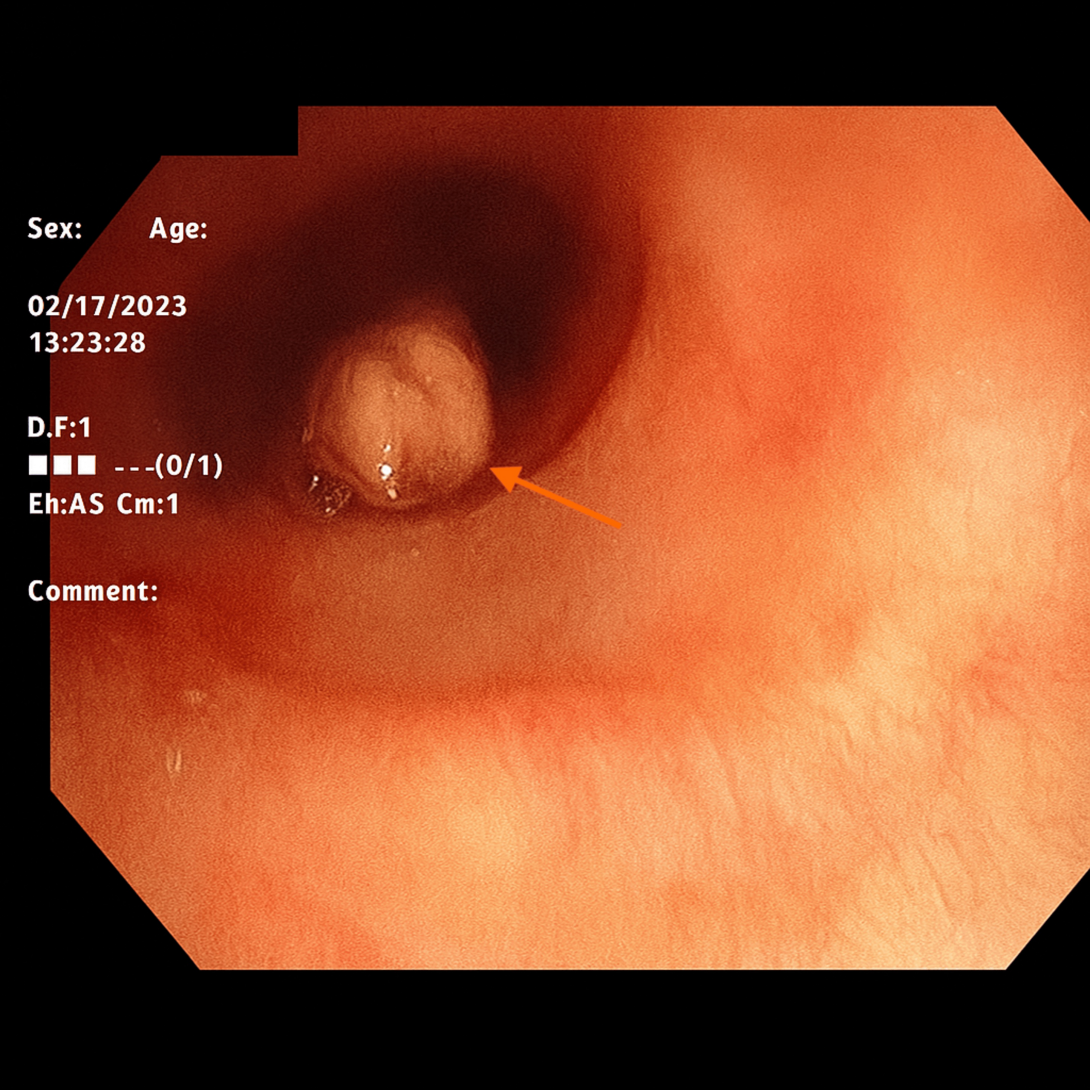
Fiberoptic bronchoscopy demonstrated an obstructing mass (orange arrow) at the left upper lobe takeoff.

Fiberoptic bronchoscopy was performed showing a large obstructing elongated mass at the LUL takeoff (orange arrow), which, after debulking, was seen to extend from the lingula (Figure 3).

**Figure 4:**
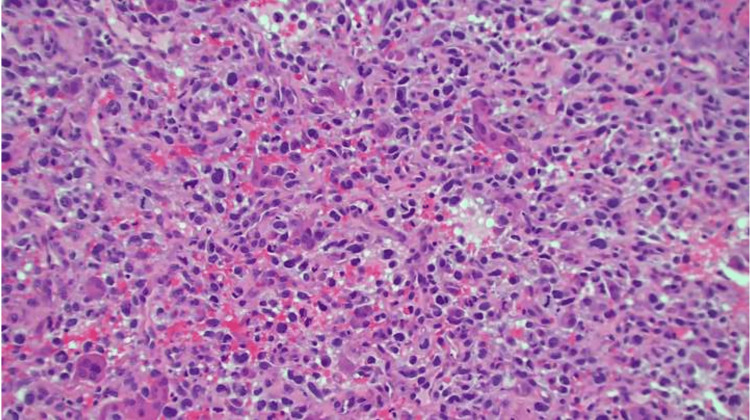
40x magnification view of biopsy of the lesion, consistent with de-differentiated LMS. LMS: Leiomyosarcoma

Pathology of the biopsied lesion showed a high-grade malignant neoplasm with blood clots that were weakly positive for *GATA3*, negative for *SMA, desmin, AE1/A3, CAM 5.2, TTF-1, CDX2, ER*, and *pax8*, consistent with metastatic de-differentiated LMS (Figure 4).

Given rapid progression of the metastatic disease with persistent bilateral air leaks, she was deemed to be too unstable for operative intervention. An attempt was made at providing systemic therapy with one cycle of doxorubicin 60 mg/m² intravenously administered while inpatient. Her renal and hepatic function was within limits tolerated for administration of this therapy (aspartate aminotransferase 39 U/L (reference range: 10-40 U/L); alanine aminotransferase 14 U/L (reference range: 7-55 U/L); total bilirubin 0.6 mg/dL (reference range: 0.2-1.2 mg/dL); alkaline phosphatase 60 U/L (reference range: 45-150 U/L); creatinine 1.01 mg/dL (reference range: 0.6-1.1 mg/dL). After receiving this chemotherapy, her condition and respiratory status continued to deteriorate with enlarging bilateral SPTX and respiratory failure requiring intubation. The patient was eventually transitioned to comfort measures and passed away peacefully thereafter.

## Discussion

To our knowledge, this is one of the few cases of bilateral spontaneous pneumothorax in a patient with uterine LMS, underscoring the need for early recognition and treatment.

Uterine LMS is an infrequent and aggressive malignancy that originates from the smooth muscle cells of the uterine tissue, and LMS is well known to metastasize. Lung (67.7%) is the most common site of metastasis, followed by cranial/intracranial (16.2%), skin/soft tissue (15.3%), and bone (13.8%) [2]. Although the time to first metastasis is highly variable in uterine LMS, the lung and peritoneum have been demonstrated to be the earliest sites of metastasis [2]. EBM is a tumor that invades the proximal central bronchus or subsegmental bronchi and has lesions histologically consistent with the primary tumor. Incidence of EBM has been reported around 28%, and this condition is often accompanied by respiratory symptoms [3,4]. Malignancy-related SPTX is uncommon, occurring in 0.05 to 1% of all SPTX, with a 1.9% frequency in patients with soft tissue sarcoma [5]. LMS-associated PTX has a poor prognosis and a high recurrence rate [5].Here, we report a case of EBM of uterine LMS presenting as bilateral SPTX.

The lung is one of the most common organs in which extra-thoracic malignant tumors metastasize, but EBM is an uncommon phenomenon. The rate of EBM from extra-thoracic malignancies is estimated to occur between 2% and 50% of the time, depending on the type of original extrapulmonary solid malignancy [6,7]. However, since bronchoscopy is not performed frequently in all patients with lung malignancies, the incidence and frequency of EBM may be underestimated [5]. EBMs from non-pulmonary sites commonly form due to the infiltration of the primary malignancy in mucosal lymphatics, which then consolidate, resulting in the formation of a solid tumor causing bronchial symptoms [8,9]. Bartosch et al. have cited a 19% 5-year survival rate in uterine LMS patients with distant metastasis [2].

Kiryu et al. proposed the following developmental modes of EBM: type I, direct metastasis to the bronchus; type II, bronchial invasion by a parenchymal lesion; type III, bronchial invasion by mediastinal or hilar lymph node metastasis; and type IV, peripheral lesions extended along the proximal bronchus [6]. In their report, all cases of EBM from primary uterine carcinoma were classified as type III lesions [6]. Our patient’s case follows developmental mode type II, given extension to the LUL carina from the lingula parenchyma. To our knowledge, descriptions of pneumothorax resulting from EBM in LMS are limited to case reports. One such report by Ghosh et al. described left lung collapse, or pneumothorax, with left main bronchial involvement in a 38-year-old female with uterine LMS [1].

LMS accounts for up to 6% of SPTX cases in sarcoma patients [5]. The processes underlying malignant SPTX may include dilated alveolar rupture into the pleural space, bronchopleural fistula, or concomitant emphysematous lung disease [9]. In our patient, the endobronchial mass obstructed the airway, causing lung collapse and creating a negative pressure space in the pleura. The most common symptoms of SPTX in sarcoma patients are dyspnea (83%), chest pain (36%), cough (7%), and hemoptysis (6%), although up to 18% of patients may be asymptomatic [5]. 

Our case discusses an infrequent presentation of bilateral spontaneous pneumothorax. Imaging was primarily indicative of a unilateral focus of metastasis on the left side. It is possible that the right pneumothorax developed because of sheer force stress on the right lung, as the left lung developed atelectasis as a result of the surrounding metastatic lesion. The endobronchial lesion may have also caused diminished air entry, which, in a patient with diminished pulmonary reserve as a consequence of metastatic disease, likely led to worsening respiratory prognosis and failure. 

## Conclusions

In conclusion, reports of EBM from endometrial cancer are extremely rare. Diagnosis of etiology remains challenging due to the absence of specific clinical characteristics. In this condition, an accurate diagnosis can only be made on pathological examination along with immunohistochemistry stain. To our knowledge, this report details a rare case of bilateral SPTX from EBM secondary to uterine LMS. Given the poor prognosis and recurrence rates of LMS-associated PTX, symptoms of dyspnea, chest pain, and cough in sarcoma patients should prompt evaluation for pulmonary metastasis, especially endobronchial tumor involvement and PTX.
